# An in vivo study of the effect of c-Jun on intervertebral disc degeneration in rats

**DOI:** 10.1080/21655979.2021.1946459

**Published:** 2021-07-24

**Authors:** Ming Lei, Kangcheng Zhao, Wenbin Hua, Kun Wang, Shuai Li, Xinghuo Wu, Cao Yang

**Affiliations:** Department of Orthopaedics, Union Hospital, Tongji Medical College, Huazhong University of Science and Technology, Wuhan, China

**Keywords:** c-Jun, intervertebral disc degeneration, TGF-β

## Abstract

Intervertebral disc degeneration (IDD) has been well-recognized as one of the causes of vast lower back pain. The objective of the current study intends to elucidate the influence and regulatory molecular mechanisms of c-Jun on IDD. This study established an IDD model of Sprague-Dawley (SD) rats by needle puncture. An LV5-c-Jun lentiviral vector was constructed and injected into rats’ intervertebral disc (IVD) tissue to increase the c-Jun expression following the establishment of modeling. The pathological changes of IVD tissue structure and collagen fibers were visualized following the processes of hematoxylin-eosin (HE) staining method and transmission electron microscopy. Real-time PCR, western blot, immunohistochemistry, and ELISA assays were performed to detect the expression levels of TGF-β, TIMP-3, COL2A1, and inflammatory cytokines. The collagen fibers were arranged in parallel and the surface was smooth after *c-Jun* overexpression, whereas the collagen fibers in the control group were disorderly arranged with a rough surface. The findings indicated that c-Jun was responsible for upregulating expression levels of TGF-β, TIMP-3, and COL2A1 in the mRNA and proteins, but simultaneously downregulating expression levels of inflammatory factors IL-1β, IL-17, IL-6, and TNF-α. c-Jun overexpression produced a positive effect on IDD, inhibited inflammatory response *in vivo*, and might delay the degeneration of IVD. Thus, c-Jun may act as a novel potential agent in treating IDD.

## graphical

**Figure:**
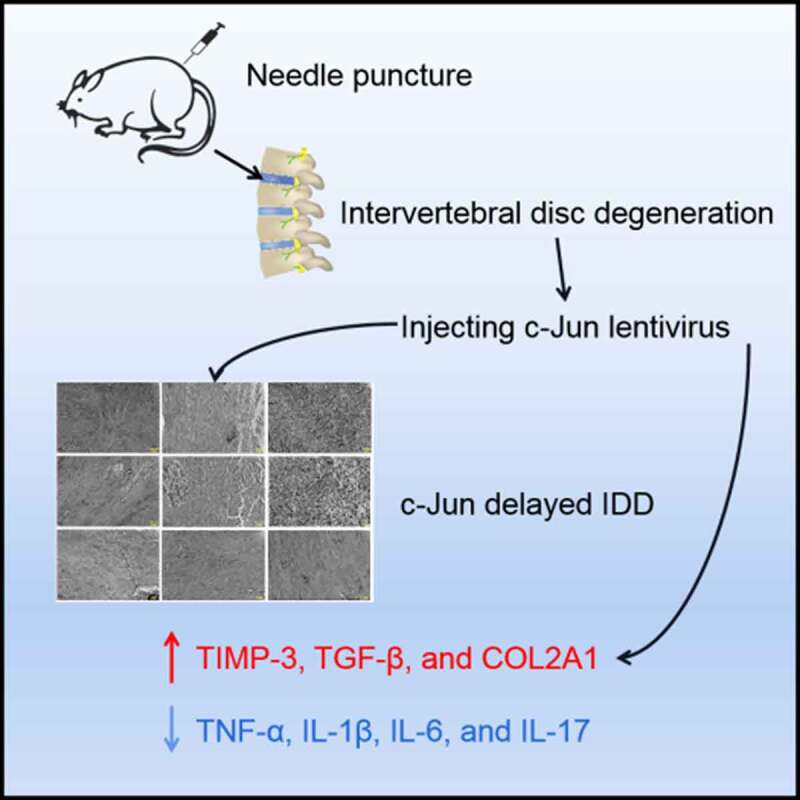

## Introduction

Lower back pain primarily concerns the degeneration of IVD which includes nucleus pulposus (NP), annulus fibrosus, and cartilage endplate. In some serious conditions, patients with disc degeneration may develop impairment in the nerve system, affecting their abilities to work, and even their quality of life [1]. It is statistically reported that over 84% of the population has experienced lumbago torture at a certain period in their lives [2]. This disorder has brought about a heavy financial burden on the social economy and costs roughly 50-100 USD billion annually, and the figure is increasing with the aging population [3]. The therapeutic strategies of IVD include pain relief by conservative management using a percutaneous intervertebral disc method, molecular therapy, and rigid fusion surgery, whereas none of these therapies has been confirmed effective and satisfactory, regardless of meeting patients’ expectations to reverse the degenerative disc [4]. The pathogenesis of IDD is complicated and influenced by both environmental and genetic factors [5]. For instance, reduced NP cell counts, imbalanced extracellular matrix (ECM) synthesis and degradation, and overexpressed inflammatory factors are ultimately correlated with the acceleration of the IDD process [6].

Type II collagen (COL2A1), aggrecan, and some components of the ECM are important for maintaining the integrity of IVD. The contents of type II collagen and aggrecan have been reported to decline and this causes a typical pathological change of IDD [7]. The imbalance between anabolism and catabolism is a vital factor to maintain components of ECM. Currently, diverse growth factors including insulin-like growth factor (IGF)-1, epidermal growth factor (EGF), and transforming growth factor (TGF)-β are recognized as small molecules playing roles in anabolic metabolism promotion [8,9]. Some molecules including tumor necrosis factor (TNF)-α, matrix metalloproteinases (MMPs), and a disintegrin and metalloproteinase with thrombospondin type I motifs (ADAMTSs) can promote catabolism [10]. The inhibition of tissue inhibitors of metalloproteinases (TIMPs) balances the catabolic activities of MMPs and ADAMTSs [11]. TNF-α can upregulate MMPs expression, causing reduction of ECM synthesis, whereas TGF-β reverses catabolism through the extracellular signal-regulated kinase 1/2 (ERK1/2) signaling pathway [12]. Furthermore, TGF-β can inhibit the expression of MMPs and delay the IDD process by promoting the synthesis of both COL2A1 and aggrecan [13]. The aggravation of IDD is the consequence of inflammatory mediators, including TNF-α, IL-1 α/β, IL-6, and IL-17 which are generated by the degenerative disc cells [14,15].

Being identified as a principal component of activator protein (AP)-1 [16], c-Jun regulation is available using diverse arrays of extracellular stimuli, pro-inflammatory cytokines, UV irradiation, oxidative and other forms of cellular stress [7,17]. Wisdom et al. (1999) have pointed out that c-Jun plays a role in fibroblast growth and apoptosis. Meanwhile, it protects cells from apoptosis induced by TNF-α, and this feature is essential for cell proliferation in cell culture [18]. Besides, through the cooperation of calcium regulatory transcription factors, the nuclear factor of activated T cell (NFAT) family with AP-1 (Fos/Jun) proteins, the inflammatory factors IL-2, IL-4, IL-8, and TNF-α can be mediated [19].

Previous reports have described that c-Jun deletion in mice causes impairment of hepatocyte proliferation and liver regeneration [20]. The absence of *c-Jun* in mice also indicated an increase of apoptosis in notochordal cells, which impedes IVD formation [21]. Overall, we hypothesized that c-Jun might play an important role in IDD. To testify whether c-Jun produces any potential influences on the gene therapy and what functions it may exert on IDD, a model of disc degeneration of SD rats was established. *c-Jun* was overexpressed in rats and histologic changes of IVD were observed by HE staining, and the changes of collagen fibers were detected using transmission electron microscopy. Subsequently, the changes of IVD-related genes in mRNA and protein expression levels were detected. It provides a new idea for the treatment of IDD.

## Materials and methods

### Experimental animals

Forty-five specific pathogen-free male SD rats were selected. The animals were 6–8 weeks of age and weighed 250 ± 20 g. All were purchased from Beijing Huakang Biotechnology Co., Ltd. The rats were fed adaptively for one week after purchase and fasted for 12 hours before experiments. The randomly divided three groups included the blank group, the empty vector transfection group, and the overexpression c-Jun transfection group, 15 in each group. Laboratory design was approved by the Animal Care and Use Committee of Huazhong University of Science and Technology.

### Lentiviral vector construction

Subsequently, c-Jun functions in rat disc degeneration were explored via constructing a lentiviral vector by c-Jun overexpression. The human *c-Jun* gene (Gene ID: 3725) was synthesized and entered an LV5 lentiviral vector, which was constructed by transferring the NotI and BamHI digested fragments. The package of lentivirus applied the following listed plasmids LV5-c-Jun, PG-p1-VSVG, PG-P2-REV, and PG-P3-RRE, which were co-transfected to the 293 T packaging cell line using fugene 6 (Promega, Madison, WI). After that, the lentiviral package was enriched by Chongqing Biomedicine Biotechnology Co., Ltd, and its experimental procedures were described previously [22]. A blank lentivirus vector (LV5-null) was used as the control and virus titer determination for both LV5-c-Jun and LV5- null lentivirus was further assessed at 1 × 10^8^ TU/mL.

### Construction of the IDD model in SD rats

The rat model of IDD was induced utilizing the needle puncture technique as previously described by Zhang et al. 2017 [23]. The animals were given 1% pentobarbital sodium at approximately 50 mg/kg for anesthesia. After successful anesthesia, a longitudinal incision along the midline was made in the abdomen. The peripheral muscles of the spine were separated to expose the IVD at level L3-6. And the cartilaginous endplate was inserted into a 21-gauge needle in parallel, 2.3 mm in depth to the disc (at L3-4, L4-5, and L5-6), rotated 180°, and kept 10 s. Depth of needle insertion was limited using a clamp as a stopper for the prevention of further insertion. The wound was sutured using a 3–0 silk thread while skin margins applying a 4–0 nylon suture. Free postoperative movement, diet, and water were available for the animals. Intramuscular injections of penicillin (8 × 10^4^ U) were given for 5 consecutive days to prevent infection. At week 4 following modeling, lentiviral were injected into the disc using a Hamilton syringe through a hole made previously with the 33-gauge needle (Hamilton Co., USA), which made the induction of degenerative disc changes unavailable according to the previously stated information [24]. The blank group received only puncture without injection. The empty vector transfection group was injected with a 5 μL LV5-null lentiviral vector. The overexpression c-Jun transfection group was injected with a 5 μL LV5-c-Jun lentiviral vector.

### Preparation of IVD tissue

Five rats in each group were killed at 4, 8, and 12 weeks post transfection. After 1% pentobarbital sodium at 50 mg/kg was injected for animal anesthesia, collection of the IVD tissue was followed which was subsequently washed with precooled PBS buffer and maintained at −80°C for further assays using western blot, qRT-PCR, and Elisa. When performing the HE staining and immunohistochemistry, IVD tissue was fixed using 4% paraformaldehyde (Solarbio, China). For transmission electron microscopy experiments, the samples were fixed with 2.5% glutaraldehyde (Solarbio, China).

### HE staining

The protocol of HE staining was similar to that used in a previous study [8]. Initially, rapid decalcification of the sections was performed in an oven with nitric acid at a low temperature and they were then embedded in paraffin following dehydration. When serial sections at the midsagittal area were obtained, they were prepared in 5-μm thickness by placing the sections in a parallel direction to the stab for staining 10 min using hematoxylin (Jiancheng, Nanjing), and washed with running water. The 1% hydrochloric acid-alcohol was used to decolorize the excess hematoxylin dye for 30 s. The samples were immersed in water for 15 min, stained with eosin solution (Beyotime, China) for 2 min, rinsed the excess dye with running water, dehydrated, and sealed with gum. Visualization and photography were performed under a microscope (OLYMPUS, Japan). The nucleus presented blue and the cytoplasm red.

### Immunohistochemistry (IHC)

The protocol of IHC was described by a previous study [8]. Following deparaffinization, the sections were incubated with 3% H_2_O_2_ (Sinopharm, China) for 10 min and 0.1% trypsin (Beyotime, China) for 20 min. Primary antibodies were subsequently incubated at 4°C overnight, using anti-TGF-β (ab31013, Abcam, USA), anti-TIMP-3 (ab39184, Abcam, USA), and anti-COL2A1 (ab34712, Abcam, USA). However, the treatment of negative control was conducted using PBS instead of the primary antibodies. The secondary antibodies used were HRP-conjugated and the section incubation was performed in a 37°C incubator for 30 min. Finally, the bound antibodies were stained with diaminobenzidine (DAB, Zhongshan, Beijing), and the sections were counterstained with hematoxylin (Jiancheng, Nanjing). Images were photographed by an inverted microscope (OLYMPUS, Japan).

### Observation of collagen fibers in IVD

The IVD tissues were fixed in the buffer containing 2.5% glutaraldehyde (Beijing Chemical Industry) for 2 h, and the solvent of l% Osmium tetrachloride (Beijing Chemical Industry) for 25–30 min following two cycles of washing with PBS. The fixed cell pellet was transferred to a sterile penicillin vial, dehydrated using acetone solution (Beijing Chemical Industry), and embedded with acetone-EPON812. The embedded nugget was cut into 1 μm thickness, dyed with methylene blue dye solution (Beijing Chemical Industry) at 60°C for 30 s, rinsed carefully with distilled water, and dyed with a composite dye containing 0.25% sodium borate and 0.5% basic fuchsin (Beijing Chemical Industry) for 10 s. After rinsing and drying, observation of the cells was made using a microscope and the location of the next thin section was marked. Ultrathin slices at 50 nm were taken, vertically immersed in a 0.45% Fonnvar solution (Beijing Chemical Industry) with a clean glass slide. After the slide surface covered with a thin film, a small piece of copper mesh was placed on the film, covered with a sealing film, removed from the glass slide, placed the ultra-thin slices on the copper mesh (on the side with the film), and then put them together into a plate. Uranyl acetate and lead dyes (Beijing Chemical Industry) was applied for staining at room temperature for 10 and 12 min, respectively, following sample cell observation under a transmission electron microscope (Philips, Netherlands).

### Western blot analysis

Total protein was extracted using RIPA lysis buffer from NP tissues followed by protein concentration measurement using an enhanced BCA Kit (Takara, Japan). The protein separation at 60 μg/lane was processed by running SDS-PAGE gel electrophoresis (Jinruisi, China) and then transferred onto a PVDF membrane (Jinruisi, China). Primary antibodies (1:1 000) were added, followed by the supplement of secondary antibodies HRP-conjugated at 1:5 000 (A0208, Beyotime, China). Chemiluminescence was detected for protein visualization (Thermo Scientific, Waltham, USA). The ImageJ 1.8.0 software (NIH) was employed and the protein expression was semi-quantified by densitometry. The findings were expressed relative to β-actin. The applied primary antibodies were presented as follows: anti-c-Jun (ab40766, Abcam, USA), anti-COL2A1 (ab34712, Abcam, USA), anti-TGF-β (ab31013, Abcam, USA), anti-TIMP-3 (ab39184, Abcam, USA), and anti-β-actin (Abcam, ab8227).

### Real-time PCR analysis

We performed real-time PCR analysis on an ABI7500 instrument (Life Technologies, Gaithersburg, MD, USA). All reaction systems and procedures of the qRT-PCR assay were performed using TB Green Premix Ex Taq II (Takara, Japan) as per the description of the manufacturer’s protocol. The relative expression levels were ultimately calculated as per the 2^−ΔΔCT^ method. c-Jun: sense-5’-

ATCGCTGCCTCCAAGTGC-3’; antisense-5ʹ- CTGTGCCACCTGTTCCCTG-3’ *TGF-β*: sense-5’-CGTGGAGGGGAAATTGAGG-3’; antisense-5’-GCCATGAGAA

GCAGGAAAGG-3’. *TIMP-3*: sense-5’-CGCAAGGGGCTGAACTATC-3’; antisense-5’-GGCGTAGT-GTTTGGACTGGTAG-3’. *COL2A1*: sense-5’-GCTCCCA

GAACATCACCTACC-3’; antisense-5’- CAGTCTTGCCCCACTTACCG-3’. *TNF-α*: sense-5’-CACGCTCTTCTGCCTGCTG-3’; antisense-5’-GGCTTGTCACTCGGGG

TTC-3’. *IL-1β*: sense-5’- AAATGATGGCTTATTACAGTGGC-3ʹ; antisense-5’-CTTGCTGTAGTGGTGGTCGG-3’. *IL-6*: sense-5’-CACTGGTCTTTT-

GGAGTTTGAGG-3’; antisense-5’- TGGGTCAGGGGTGGTTATTG-3’. *IL-17*: sense-5’-CTCTGTCCCCATCCAGCAAG-3’; antisense-5’-GTGGACAATCGGGGT-

GAC-3’. *β-actin*: sense-5’- TGACGTGGACATCCGCAAAG’; antisense-5’-CTGGA-

AGGTGGACAGCGAGG-3’.

### ELISA assay

The tissues were cut into fine fragments and 1 mL of RIPA lysate was added to every 100 mg of tissues and ground with a grinder. The homogenate was immediately placed into a new 1.5 mL centrifuge tube and centrifuged at 12,000 rpm 4°C for 5 min. The supernatant was absorbed for subsequent ELISA detection. Expression levels of inflammatory factors TNF-α, IL-1β, IL-6, and IL-17 were separately measured applying ELISA kits (Bluegene, Shanghai, China) following the instructions of the manufacturer. The absorbance value (OD value) was measured and recorded at 450 nm, based on which, the corresponding concentration was determined and multiplied via a dilution factor.

### Statistical analysis

All data were statistically analyzed using the SPSS 23.0 software (SPSS Inc., USA). The results were subsequently presented as mean ± standard deviation (SD). The experiments were replicated five times independently. Differences in data were analyzed applying the one-way analysis of variance (ANOVA) and post hoc Tukey’s test. *p* values < 0.05 were considered statistically significant.

## Results

### C-Jun expression in NP tissue

**Figure 1.**
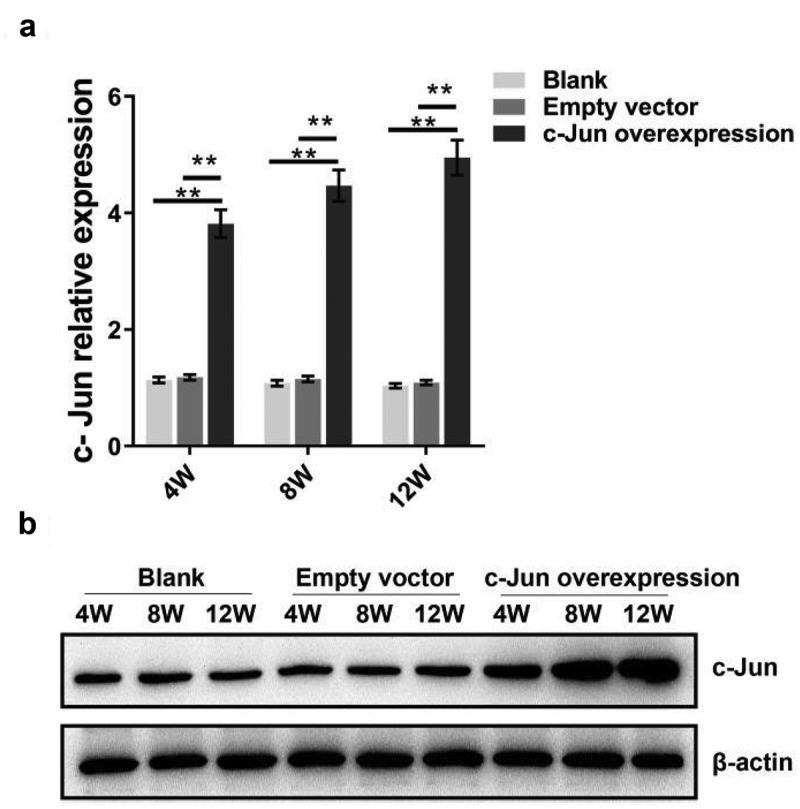
*C-Jun* expression in intervertebral disc tissue at 4, 8, and 12 weeks following the establishment of disc degeneration model. (a) The mRNA expression of *c-Jun* was subjected to qRT-PCR. (b) Protein levels of c-Jun were detected by western blot. Pairwise data analysis employed one-way ANOVA followed by post hoc Tukey’s test (mean ± SD, n = 5). ‘**’ *p* < 0.01

To explore the role of c-Jun in IDD, a model of disc degeneration of SD rats was established. The histological changes and expression levels of IVD-related genes and proteins were detected after overexpressing *c-Jun*. After lentivirus infection, qRT-PCR and western blot assays were employed to determine c-Jun expression. The expression level of *c-Jun* in the overexpression group was higher than the blank and empty vector groups following the qRT-PCR results (Figure 1a). Moreover, the expression level of *c-Jun* was indicated an increase with the passage of time. Consistent expression tendancy of c-Jun protein was revealed with that of mRNA (Figure 1b). c-Jun was successfully transfected into the NP tissue according to western blot and qRT-PCR assays.

### Histological changes and collagen expression in degenerated IVD

**Figure 2.**
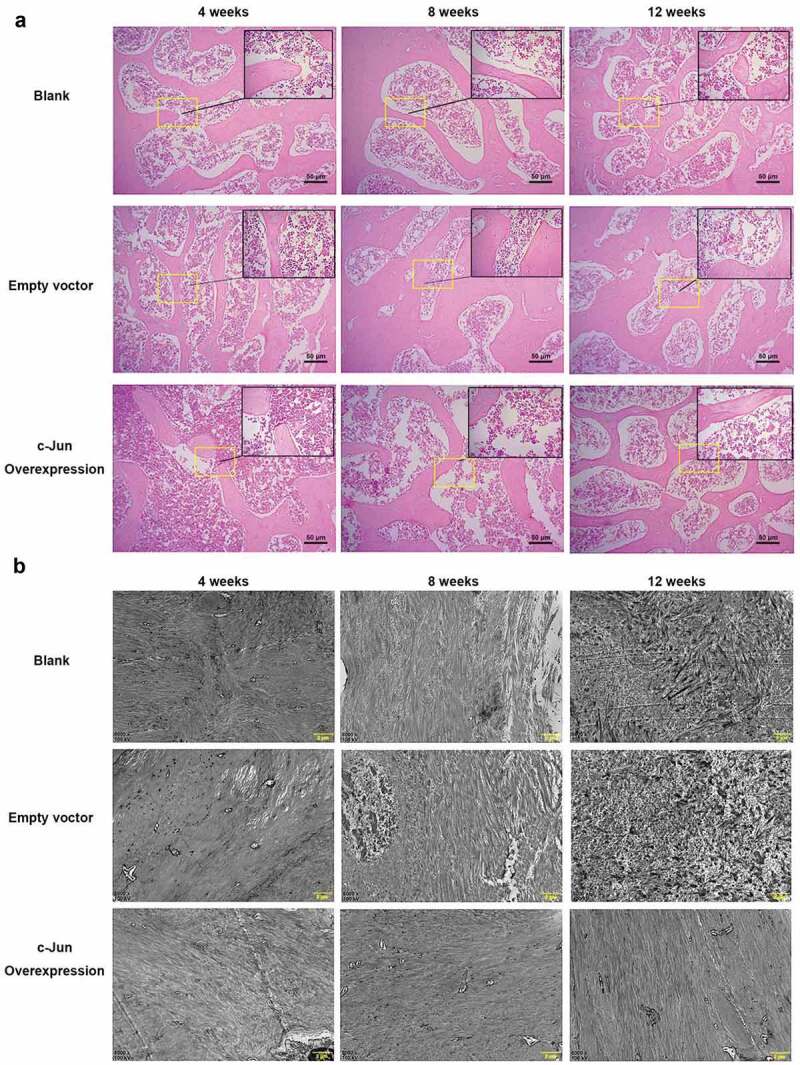
The histological structure of intervertebral disc tissue in rats was influenced by c-Jun overexpression at 4, 8, and 12 weeks after establishing models of disc degeneration. (a) Histological changes were visualized via HE staining. The scale bar was 50 μm, magnification, 100 × . (b) Collagen fiber was observed using transmission electron microscopy. The scale bar was 2 μm at a magnification of 8000 ×

After overexpressing *c-Jun*, results of HE staining (Figure 2a) showed that in the model of IDD rats, NP boundary was unclear at week 4, and a change of degeneration could be found at the center. At week 8, NP boundary turned less clear, the central degeneration-like change was more obvious, and the cell density was lower. At week 12, the NP was more indistinct, the central denatured changes were more obvious, and the cell density was further reduced. Moreover, no significant difference was revealed by pairwise comparison of the overexpressed c-Jun and the control. Further transmission electron microscopy showed that collagen fiber was arranged in parallel with a smooth surface in the *c-Jun* overexpression group, while the collagen fiber in the blank and empty vector groups was irregularly arranged with a rough surface. These results indicated that overexpression of c-Jun delayed disc degeneration (Figure 2b).

### The expression of TGF-β, TIMP-3, and COL2A1 following overexpressing c-Jun

**Figure 3.**
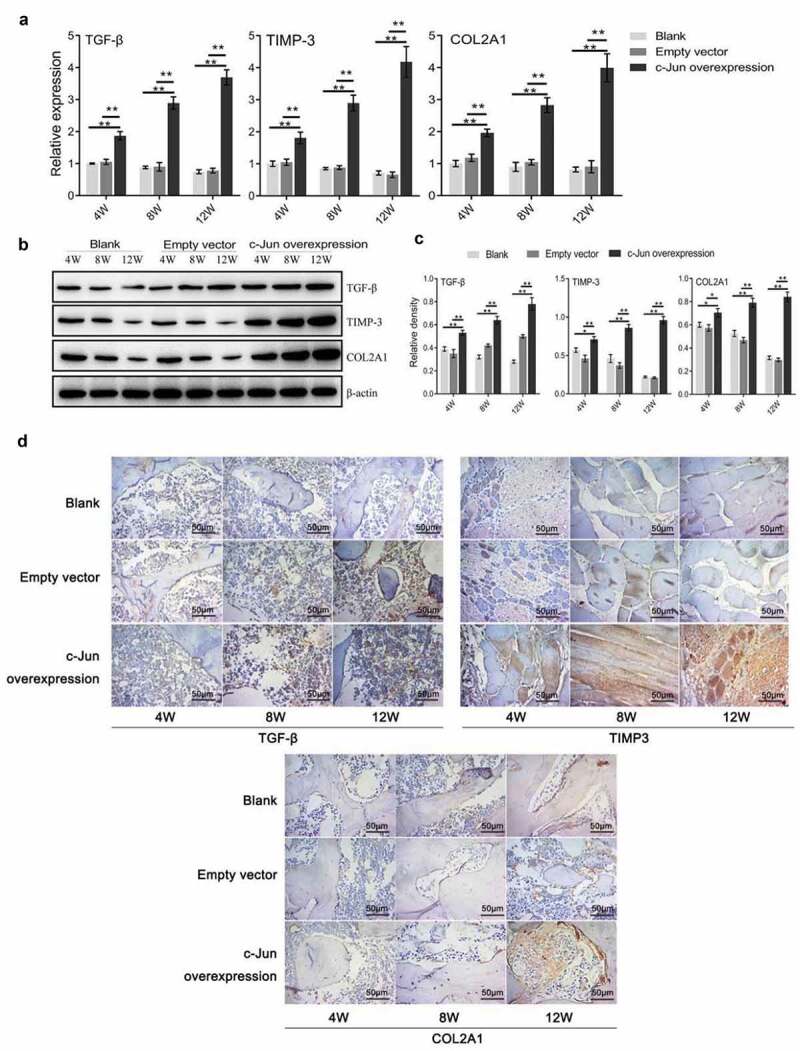
Expressions of TGF-β, TIMP-3, and COL2A1 in disc degeneration tissue were increased after overexpressing c-Jun. (a) The mRNA expressions of *TGF-β, TIMP-3*, and *COL2A1* were detected using qRT-PCR assays. (b) The protein expressions of TGF-β, TIMP-3, and COL2A1 were determined using western blot assays. (c) Relative density of TGF-β, TIMP-3, and COL2A1 in (B) was analyzed by Image J. (d) The protein expressions of TGF-β, TIMP-3, and COL2A1 were assumed by immunohistochemistry. Scale bar, 50 μm, magnification, 400 × . One-way ANOVA followed by post hoc Tukey’s test was used for data analysis (mean ± SD, n = 5). ‘*’ *p* < 0.05 ‘**’ *p* < 0.01

To investigate the role of c-Jun in IDD, we detected the expression levels of genes associated with ECM synthesis of IVD. The results of qRT-PCR indicated that the expression levels of *TGF-β, TIMP-3*, and *COL2A1* were markedly increased in the c-Jun overexpression group compared with the control group, and mounted up continuously with transfection time (Figure 3a). Western blot results were in agreement with those of the qRT-PCR (Figure 3b, c). IHC results revealed that the three proteins were highly expressed in the overexpressed group (Figure 3d). These data demonstrated that c-Jun was positively associated with disc degeneration through mediating the IVD-related gene expression.

### Detection of IL-1β, IL-17, IL-6, and TNF-α expression

**Figure 4.**
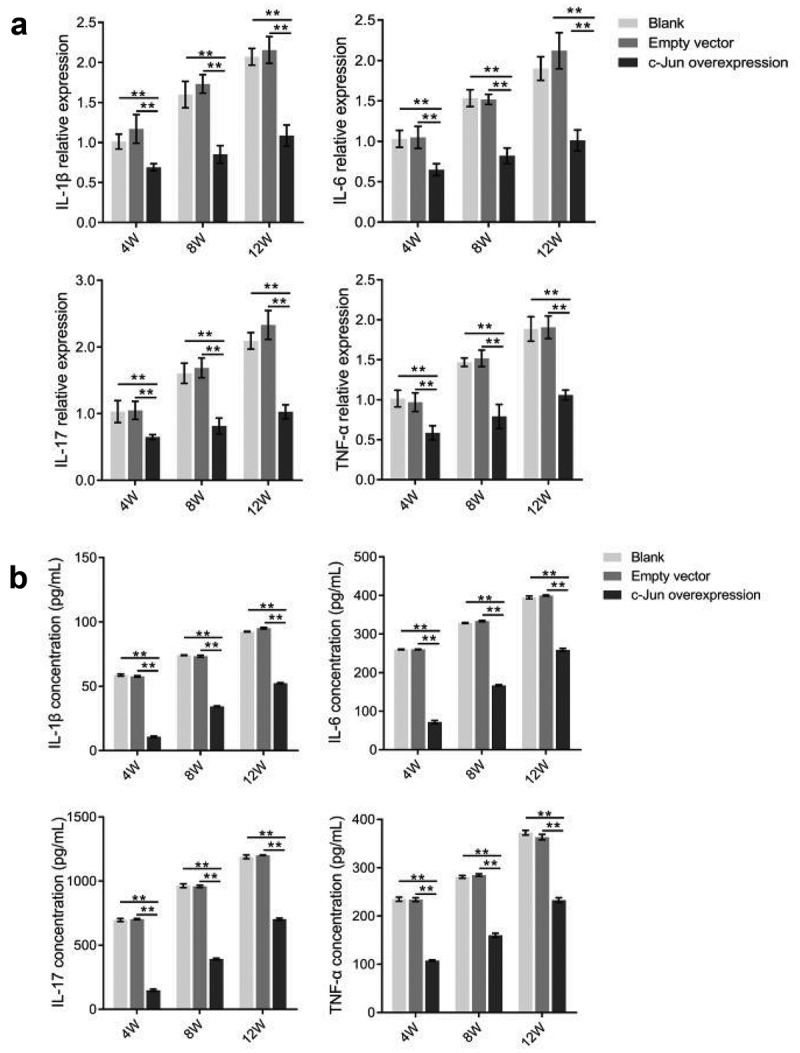
The expressions of TNF-α, IL-1β, IL-6, and IL-17 in disc degeneration tissue were decreased after overexpressing c-Jun. (a) The mRNA expressions of *TNF-α, IL-1β, IL-6*, and *IL-17* were determined using qRT-PCR. (b) The protein levels of TNF-α, IL-1β, IL-6, and IL-17 were detected using Elisa. Data analysis employed the one-way ANOVA followed by post hoc Tukey’s test (mean ± SD, n = 5). ‘**’ *p* < 0.01

To further explore the function of c-Jun, we detected the expression levels of *IL-1β, IL-17, IL-6*, and *TNF-α* genes using real-time PCR (Figure 4a). *IL-1β, IL-17, IL-6*, and *TNF-α* expression levels were markedly lower in the c-Jun overexpression group. Notably, the expression level was the highest revealed at the 12th week. Further, the expression levels of inflammation-related factors were examined by ELISA (Figure 4b). This consistent result with gene expression demonstrated that the expression levels of IL-1β, IL-6, and TNF-α could be dramatically decreased in the overexpressed c-Jun group, while increased as the transfection time prolonged. In conclusion, c-Jun downregulated the expression of inflammatory factors in mRNA and protein levels, which might delay the inflammatory response of disc degeneration.

## Discussion

Notochord cell apoptosis was increased but the disc formation was impaired in *Jun*-deficient mice [21]. In herniated discs, c-Jun immunoreactivity in disc cell clusters may demonstrate that it acts as an active transcriptional site in disc tissues [25]. Disc cell clusters were reported that they are responsible for producing specific ECM components and may function in repairing damaged IVD tissues [26]. In this study, an IDD rat model was constructed and lentivirus-packaged c-Jun was injected into the rats in vivo to observe histological changes and distribution of collagen fiber in IVD tissues. The results of *c-Jun* overexpression revealed that disc degeneration was delayed in rats, and collagen fiber was parallelized and its surface was smooth comparing the blank and empty vector groups (Figure 2b). Collagen fiber represents the main component of ECM, and it is important for maintaining the integrity of IVD [3]. c-Jun and its dimerization partners are subject to incredible diversification with multiple biological functions including Jun-Jun, Jun-ATF, and Jun-Fos dimmers [27]. It regulates gene expression and cellular function by forming these dimeric complexes characterized by high affinity and sequence-specific DNA binding activity [27]. In combination with the promoters of many genes, c-Jun regulates gene transcription functioning as a transcription factor. Previous studies have suggested that the heteromeric complex of Smad3 and Smad4 can induce transcriptional activation of the TGF-β response utilizing synergistic interaction with c-Jun/c-Fos locating at the binding site of the collagenase I promoter AP-1 [28,29]. TIMP-3, TGF-β, and COL2A1 are known to delay and repair IDD, assisting in ECM synthesis [5,30]. TGF-β has indicated a role in increasing COL2A1 expression in adolescent NP cells compared with adult degenerated NP cells [31]. TIMPs maintain balance of both specific inhibitors MMPs and balance between anabolism and catabolism of ECM by inhibiting MMPs [32]. Moreover, TGF-β1 increased the expression of TIMP-3 and promoted NP cell proliferation in human IDD [33]. Our previous cell experiments showed that overexpression of c-Jun markedly upregulated TIMP-3, TGF-β, and COL2A1 levels [7]. This study confirmed that the expression levels of TIMP-3, TGF-β, and COL2A1 were substantially higher in rat disc tissues following *c-Jun* injection (Figure 3). Furthermore, their expression level in the overexpressed c-Jun group was increased over time, while those in the non-transfected and empty vector groups were decreased gradually. These results highlighted that c-Jun probably delayed disc degeneration through up-regulating TGF-β. This also implied that c-Jun was positively associated with disc degeneration progression.

As one of the extracellular stimuli, the pro-inflammatory cytokine can regulate c-Jun expression [34–36]. In NP cells, IL-17A can mediate the inflammation of IVD through the p38/c-Fos and JNK/c-Jun signaling pathways [36]. Many researchers have reported that inflammatory mediators play a critical role in IVD, they contribute to the processes of disc degeneration [37]. Compared with normal disc tissues, the expression levels of inflammatory factors were greatly increased in the degenerated disc tissue. Meanwhile, other researchers have identified that the levels of IL-1β, IL-6, IL-17, and TNF-α have a positive relationship with the degree of disc degeneration [38,39]. The function and molecular mechanisms of c-Jun in IDD inflammation are poorly understood. c-Jun in our study produced anti-inflammatory effects on the degenerated disc tissues. Both ELISA and qRT-PCR analysis showed that the expression levels of inflammatory cytokines TNF-α, IL-1β, IL-6, and IL-17 were inhibited after overexpressing *c-Jun* in the disc degeneration rats. As the injection time prolonged, an increase in the expression of inflammatory cytokines was visualized as well, while the c-Jun overexpression group was always lower than the empty vector group (Figure 4). This indicated that c-Jun might inhibit inflammation and reduced the progression of IDD.

IDD has emerged as a major concern in the public healthcare domain. Meanwhile, gene therapy has turned into a potential therapeutic method to retard disc degeneration, which can be achieved either by employing vectors carrying associated genes in vivo or by using target cells in vitro [40]. Lentivirus can integrate stable genes into host genome and it is characterized by favorable features of cell infection, division, and non-division. With a wide range of tissue tropisms, and nonexpression of viral proteins after transduction, it is considered to be an efficient and stable vector that is extensively applied in gene therapy of IVD degeneration [41]. Zhang et al. (2018) reported that the previously described advantages of lentivirus including persistent expression and immunologic tolerance contribute a lot to gene modification of vertebral discs composition [42]. In an animal model of IDD, co-transduction of lentiviral plasmid TGFβ3-P2A-CTGF-T2A-TIMP1 into the IVD can delay degeneration through increasing collagen type II and aggrecan synthesis [42]. As for the species selection of animal models, primates are the closest to humans in terms of physiology. But due to the limitations of ethical and economic factors, the model of induced disc degeneration made by rats or rabbits is the most widely used [43]. The present experiments confirmed that the transduction of lentivirus-mediated c-Jun was effective in delaying IDD in the rat model. Importantly, there was no reported side effects in rats during the experiment processes. However, clinical cases are more complicated than animal models, which may produce great risks of side effects if applied clinically. Of note, no distinct differences in histological appearances between the c-Jun-transfected group and the control group were observed, although transmission electron microscopy displayed changes in collagen fibers. Additionally, since the c-Jun knockout experiments have not been conducted yet, the mechanisms of c-Jun on IDD delay needs further exploration.

## Conclusion

Taken together, this study demonstrates that c-Jun affects IDD treatment positively. It can slow down the progression of IDD, promote the expressions of TIMP-3, TGF-β, and COL2A1, and inhibit the inflammatory response of cytokines TNF-α, IL-1β, IL-6, and IL-17 via downregulating their expression levels. It is suggested that c-Jun may produce a good therapeutic effect on the treatment of disc degeneration.
